# Welcome to Volume 5 of *Concussion*

**DOI:** 10.2217/cnc-2020-0001

**Published:** 2020-02-06

**Authors:** Kimberley Ndungu, Lauren Pulling, Jennifer Straiton

**Affiliations:** 1 Future Science Group, Unitec House, 2 Albert Place, London, N31QB, UK

To all of our readers, Happy New Year and welcome to the first issue of Volume 5 of *Concussion*. As we celebrate our 5th birthday as a journal, we are proud to maintain our commitment to publishing the key advances in clinical and translational research across this niche, yet fast paced, area of research.

In this Foreword, we take a look back at some of our content highlights from 2019, as well as introduce our new resource for concussion researchers: Concussion Zone [1]. We also provide an update regarding the work of our parent, The Drake Foundation [2], on behalf of which the journal is published; looking at the projects they are currently funding in order to help further concussion research.

## Content highlights of 2019

The standard of articles published over the past year has remained at the high quality for which we are known and we would like to thank all of our authors and reviewers, each of whom has worked to ensure that all work follows the highest of standards.

Our most read article of the year comes from a research team led by Melissa Hunfalvay of RightEye LLC (MD, USA) and describes their novel eye-tracking tests, which measure both horizontal and vertical saccades to create an objective and quantifiable method of differentiating between severities of traumatic brain injury [3]. An altmetric score of 58 puts this article in the top 5% of all research outputs scored by the metrics system and in the 95th percentile of articles of the same age. A follow-up article from this research group was also published in this issue and we recommend you look out for it.

A hot topic of the year has been concussion in school- and college-aged adolescents. First, we had a research article written by Arthur Maerlender *et al.*, reporting on a training model to be given to school personnel that will improve knowledge on how to manage concussion in a student [4]. Based on ten key knowledge competencies, the training method gained positive feedback from those involved in the study, with many reporting the competencies to be informative and useful. For anyone who works in concussion management, this is a key read.

Following the theme of concussion in young people, we have another research article, this time with a focus on college students. In this article, the research team, led by Zachary W Bevilacqua (Indiana University, ID, USA), present a new approach to monitor concussed students and determine when they are recovered enough to re-enter the academic setting [5]. This concept, commonly referred to as return-to-learn, has been at the forefront of concussion research and as all student-athletes are first and foremost students, a greater understanding is of vital importance.

Our final paper to mention comes from Stanford’s (CA, USA) Angela Lumba-Brown and colleagues. In their perspective article, they provide an overview of commonly used post-concussion symptom-rating scales and determine the representation of different subtype-directed symptomatologies [6]. They found that overall, the percentage of symptoms representing each subtype was low, though ocular-motor, vestibular and cervical strain-associated conditions were the least represented. This under-representation of key symptoms marks a clear unmet need in the field of concussion research.

## Editorial board

To our Editorial board, we thank you for your continued input, be it in an ambassadorial, advisory or authorship role. Our international board provides valuable assistance and advice that facilitate the publication process; we look forward to working together in the coming year.

On this note, if you are interested in joining our advisory board or wish to provide feedback or suggestions for the journal, please do not hesitate to get in touch; we value any and all input that can contribute to the growth and development of the journal.

## Concussion zone

Launched in September this year, Concussion Zone [1] is a new community site covering the latest news, expert opinions and insights into concussion and traumatic brain injury. On Concussion Zone you can find content and discussion across hot topics in medical research and practice, sport, medico-legal issues and regulation and guidance.

By following channels on the site, such as assessment and rehabilitation, imaging or sports and exercise medicine, you can keep up to date on your specific topics of interest. Further, in the events channel, you can find our Editor’s reports from relevant conferences.

Registration to Concussion Zone is free and will provide you with exclusive access to editorials, opinion pieces and interviews from experts within the field of traumatic brain injury, plus optional newsletters via email to keep you up to date with the latest research.

Some of our top pieces of content since our launch include a news piece following the publication of the highly anticipated FIELD study, which reported that professional football (soccer) players are 3.5-times more likely to die due to dementia and over five-times more likely to die due to Alzheimer’s disease [7] and an editorial in which Claire Baker (Imperial College London, UK) discusses how on-board sensors could be utilized to reduce the effect of head trauma and gives insight into the work being conducted in the AutoTRIAGE project [8].

If you have any ideas on what you would like to see on Concussion Zone or are interested in writing for the site, then please get in touch.

## Update from The Drake Foundation

2019 was a big year for The Drake Foundation, with the kick-off of several new projects, as well as progress updates from several others.

In January came the announcement of a new research study to evaluate concussion in Premier League football (soccer) [9]. The study is being led by Tony Belli and Patrick O’Halloran (both University of Birmingham, UK), with support from the Premier League and funding from the Foundation. Over the 2018/19 and 2019/20 seasons, saliva and urine samples will be collected from players who sustain a head injury, as well as noninjured controls. These samples will then be tested using the Birmingham Concussion Test to examine the potential of microRNAs as biomarkers of brain injury. The hope is that the test could 1 day be used pitch-side, with the potential to assist in return-to-play decisions and concussion diagnoses across sport, the military and other frontline settings.

This year has also seen the start of recruitment for the HEADING (Health and Ageing Data IN the Game of football) study [10], which will recruit 300 former elite football (soccer) players in order to examine the link between a history of concussion and heading the ball with neurodegenerative disease and long-term cognitive function.

Another exciting new addition to The Drake Foundation’s research portfolio was launched in 2019: The Drake Football Study (DFS) [11]. The DFS comes as a result from a groundbreaking collaboration between the Foundation (as founding funders) and FIFPRO, the PFA and football players unions across Europe. Researchers will measure the health of professional footballers; a population that has been shown to be more likely to experience problems with their mental, musculoskeletal, neurocognitive and cardiovascular health. Until now, available evidence has been established principally from cross-sectional studies focusing on a specific health domain in either active or retired players. The DFS is the first prospective cohort study that monitors the health of professional footballers across domains (mental, musculoskeletal, neurocognitive and cardiovascular) before, during and after their retirement out of sport. This will hopefully fill the gaps in our knowledge around the onset and progression of several health conditions, which will help equip clubs and medical teams to support future players both in their careers and throughout retirement.

Outside the research funding, in November 2019 was the 4th Annual UK Sports Concussion Research Symposium, this year jointly hosted by The Drake Foundation, Football Association, Rugby Football Union, England and Wales Cricket Board and British Horseracing Authority. This year’s symposium marked a significant year in the concussion field, with media attention and public interest in the long-term effects of sports concussions continuing to grow, particularly around the publication of the first results from the FIELD study. It was fantastic to hear progress from speakers in concussion research, sporting bodies and technology development, with talks centering on diagnostic and recognition tools, understanding of recovery and medium and long-term consequences of head injury. For a full rundown of the symposium, take a look at the conference report [12] on Concussion Zone.

As always, 2020 looks set to be another busy year, with results and publications expected across a number of the Foundation’s projects and the launch of more new initiatives across science, sport and society. For more information and the latest news from The Drake Foundation, visit our website [2].

## Conclusion

Our readers remain central to the success of our journal and we welcome any feedback. Please do not hesitate to contact us with any suggestions for what you would like to see featured or any article proposals of your own. We welcome a wide range of unsolicited article proposals, so please do get in touch for more information.

We would like to finish by once again thanking all of the authors and reviewers who helped to make Volume 4 possible; we hope to continue to build on this success and look forward to another great year.
